# Regulating compassion: an overview of Canada's federal medical cannabis policy and practice

**DOI:** 10.1186/1477-7517-5-5

**Published:** 2008-01-28

**Authors:** Philippe G Lucas

**Affiliations:** 1 Vancouver Island Compassion Society. 130-2017a Cadboro Bay Rd. Victoria, BC, Canada; 2 Studies in Policy and Practice, Faculty of Human and Social Development, University of Victoria, PO Box 1700, Stn, CSC 3800 Finnerty Road, Victoria, BC V8W 2Y2, Canada; 3 Center for Addictions Research of British Columbia, University of Victoria. PO Box 1700 STN CSC, Victoria BC, V8W 2Y2, Canada; 4 Canadians for Safe Access. 130-2017a Cadboro Bay Rd. Victoria, BC, Canada, DrugSense, 14252 Culver Drive #328, Irvine, 92604-0326, Canada; 5 1104 Topaz Ave, Victoria, BC, V8T 2M7, Canada

## Abstract

**Background:**

In response to a number of court challenges brought forth by Canadian patients who demonstrated that they benefited from the use of medicinal cannabis but remained vulnerable to arrest and persecution as a result of its status as a controlled substance, in 1999 Canada became the second nation in the world to initiate a centralized medicinal cannabis program. Over its six years of existence, this controversial program has been found unconstitutional by a number of courts, and has faced criticism from the medical establishment, law enforcement, as well as the patient/participants themselves.

**Methods:**

This critical policy analysis is an evidence-based review of court decisions, government records, relevant studies and Access to Information Act data related to the three main facets of Health Canada's medicinal cannabis policy – the Marihuana Medical Access Division (MMAD); the Canadians Institute of Health Research Medical Marijuana Research Program; and the federal cannabis production and distribution program. This analysis also examines Canada's network of unregulated community-based dispensaries.

**Results:**

There is a growing body of evidence that Health Canada's program is not meeting the needs of the nation's medical cannabis patient community and that the policies of the Marihuana Medical Access Division may be significantly limiting the potential individual and public health benefits achievable though the therapeutic use of cannabis. Canada's community-based dispensaries supply medical cannabis to a far greater number of patients than the MMAD, but their work is currently unregulated by any level of government, leaving these organizations and their clients vulnerable to arrest and prosecution.

**Conclusion:**

Any future success will depend on the government's ability to better assess and address the needs and legitimate concerns of end-users of this program, to promote and fund an expanded clinical research agenda, and to work in cooperation with community-based medical cannabis dispensaries in order to address the ongoing issue of safe and timely access to this herbal medicine.

## 1. Introduction

Although modern medicine has only recently begun to rediscover the therapeutic potential of cannabis, written records of medical use date back thousands of years. The first known mention of cannabis as a medicine appears in the world's oldest known medical text, the *Pen Ts'ao Ching*. Apparently composed by Emperor Shen-Nung around 2800 B.C., the oldest written copy dates back to the first century and suggests that cannabis may be useful in treating hundreds of conditions, including rheumatism, menstrual fatigue, and malaria [1,2]. During the 17^th ^and 18^th ^centuries, western medical practitioners learned of its many therapeutic properties from the traditional practices of China and India. By the 19^th ^century, cannabis was common in many widely used pharmaceutical preparations [3], and well-known drug companies like Merck, Burroughs-Wellcome, Bristol-Meyers Squibb, Parke-Davis and Eli Lilly manufactured cannabis-based treatments for pain, digestive conditions, asthma, sleeplessness and depression. With the advent of injection drugs and semi-synthetic analgesics such as acetylsalicylic acid, cannabis fell out of popular use in Western medicine early in the 20^th ^century [4,5].

In Canada, this coincided with the rise of a moral entrepreneur named Emily Murphy who, in 1916, became the first female judge in the British Empire. Four years later MacLean's Magazine published a series of Murphy's sensationalist and xenophobic articles on opium and cannabis use. This not only prompted major legal reform in regards to drug enforcement, but also led to the addition of cannabis to Canada's list of prohibited substances in 1923 without any significant public debate [6,7]. Over the next two decades, the international implementation of cannabis prohibition effectively put an end to nearly all research into its medicinal use.

With the popularization of cannabis as a recreational drug in the 1950's and 1960's, scientific research into its potential harms and therapeutic uses slowly re-emerged [8]. Despite the continued prohibition of its recreational use in most of the world, three countries – the United States, Holland and Canada – have allowed very limited patient access to cannabis through centralized national medical cannabis programs.

This paper examines the origin and evolution of three major components of the Canadian federal medical cannabis program: 1) Health Canada's Marihuana Medical Access Division; 2) the Canadian Institute of Health Research Medical Marijuana Research Program; and 3) Prairie Plant Systems and the federal production contract. In addition, this overview will also examine a community-based alternative to the centralized government monopoly on the production, research, and distribution of cannabis: Canada's informal network of medical cannabis dispensaries.

### 1.1 Court-Ordered Compassion: Canada's Federal Medicinal Cannabis Program

In 1999 Health Canada initiated a centralized federal medicinal cannabis program in response to an Ontario court challenge. This 1998 court case focused on Jim Wakeford, a person living with HIV/AIDS who faced cannabis possession and cultivation charges for attempting to grow a supply of medical cannabis to treat symptoms of his condition. The Ontario Superior Court recognized his legal right to access cannabis without fear of arrest, and instructed Health Canada to create a process allowing for legal access to this medicine. Health Canada responded by pointing to existing legislation – Section 56 of the *Controlled Drugs and Substances Act *(CDSA) – that would grant qualified applicants a federal exemption from the section of the CDSA addressing cannabis possession (Wakeford v. the Queen, 1999).

The following year, the Ontario Court of Appeals heard the case of a man named Terry Parker, who had been charged with cannabis possession and cultivation while growing a personal supply to alleviate symptoms of his epilepsy. The appellate court struck down the Section 56 program as unconstitutional when it was revealed that the process was not subject to regulatory oversight and instead granted total discretion to approve or reject potential applicants to the Health Minister. The court also struck down Section 4 of the CDSA as it relates to cannabis possession for all Canadians, but suspended the ruling for one year in order to allow the government time to introduce fair and appropriate regulations enabling access to medicinal cannabis for those with a legitimate medical need (Parker v. the Queen, 2000). As a result of these legal challenges, the constitutional validity of Canada's drug control regulations is now legally dependent on the existence of a working federal medicinal cannabis program.

Since these initial developments Health Canada has created the *Marihuana Medical Access Division *(or MMAD, formerly known as the Office of Cannabis Medical Access, or OCMA) to act as the governing body overseeing the implementation of the *Marijuana Medical Access Regulations *(MMAR), which replaced the "Section 56" exemption process in 2001 [9].

On January 9^th ^of 2003 – in a ruling stemming from a lawsuit initiated by medicinal cannabis users and suppliers – the Ontario Supreme Court upheld the right for patients to have access to a safe, legal source of cannabis and once again found the federal program unconstitutional for creating what provincial judge Lederman called the "illusion of access." The court gave the government until July 9^th ^of the same year to put forward a legal supplier for medical users authorized under the Marijuana Medical Access Regulations (Hitzig v. the Queen 2003).

On the eve of July 8^th ^2003, with the announcement that Health Canada would soon accept written requests by federally-registered users for the cannabis being grown under contract by Prairie Plant Systems (PPS), Canada became the second nation in the world to put in place a system for access to medical cannabis through a centralized, government-administered program (the first was the U.S. Investigational New Drug (IND) program, which began supplying cannabis in 1979, but ceased taking applications in 1989). However, this did not save Health Canada's program from being found constitutionally deficient later that year. On October 7^th^, the Ontario Court of Appeals declared five specific sections of the MMAR invalid, including the restrictions on production that prevented compassion clubs from operating as legal entities:

[161] We have earlier described the ineffectiveness of the DPL (Designed Production License) provisions of the *MMAR *to ensure a licit supply to [federal license] holders. That ineffectiveness appears to stem very largely from two prohibitions in the *MMAR*. First, a DPL holder cannot be remunerated for growing marihuana and supplying it to the ATP holder. Second, a DPL holder cannot grow marihuana for more than one ATP holder nor combine his or her growing with more than two other DPL holders. These barriers effectively prevent the emergence of lawfully sanctioned "compassion clubs" or any other efficient form of supply to ATP holders. (Hitzig v Canada, 2003)

Although the Ontario Court of Appeals decision immediately struck down these five barriers, on December 17^th^, 2003 Health Canada re-instated the limits on production verbatim, defending their actions by suggesting that:

...these limits on the production of marihuana are necessary to maintain control over distribution of an unapproved drug product, which has not yet been demonstrated to comply with the requirements of the FDA/FDR; minimize the risk of diversion of marihuana for non-medical use; be consistent with the obligations imposed on Canada as a signatory to the United Nations' *Single Convention on Narcotic Drugs*...; and maintain an approach that is consistent with movement toward a supply model whereby marihuana for medical purposes would be subject to product standards, produced under regulated conditions; and distributed through pharmacies...[10]

To date, a program allowing for pharmacy-based access to medical cannabis has yet to be implemented, and by re-instating the regulations that the Ontario Court of Appeals had recently struck down, Health Canada once again brought this program into questionable constitutional standing.

Despite ongoing controversy surrounding the administration of the federal medical cannabis policy, Canadians overwhelmingly support its use. According to the Project Canada Survey Series conducted by sociologist Reginald Bibby since 1975, recent polling indicates that 93% of Canadians support the legal medical use of cannabis [11].

### 1.2 A Brief History of North America's Community-Based Medical Cannabis Dispensaries

During the late 1980's, as rates of HIV and AIDS began to rise in San Francisco, a few underground dispensaries offering a safe source of cannabis to those needing it for medical purposes were established by compassionate people living with HIV/AIDS and drug policy reform activists. With the successful passage of a state ballot initiative called "Proposition 215" in 1996, California became the first U.S. state to allow for the legal medical use and distribution of cannabis. Within a few weeks dozens of these "compassion clubs" opened, and although they often had varied policies and practices, their common goal was facilitating access to a safe supply of cannabis for medical users [12]. Since then, over 250 community-based medical cannabis dispensaries have opened up in California, and it is estimated that they currently supply over 200,000 state authorized patients [13]. Similar organizations have emerged all over the world, and in Canada and the U.S. these dispensaries remain the main source of cannabis-based medicines for therapeutic use.

There are currently seven well-established compassion clubs or societies in Canada, the oldest and largest of which is Vancouver's British Columbia Compassion Club Society (BCCCS). The BCCCS opened in 1997 and now serves over 4000 members [14]. Taking a holistic approach to health, this non-profit organization operates a Wellness Center offering alternative treatments such as Traditional Chinese Medicine, acupuncture, counseling, and herbalism at a reduced cost to members of the society. The Vancouver Island Compassion Society (VICS), which has been a registered non-profit society in B.C. since October of 1999, has used its knowledge and experience of cannabis and its therapeutic properties to implement an extensive research agenda.

Although the Canadian federal government has not legally recognized any of the nation's compassion clubs, many of these organizations have had the opportunity to inform the public debate surrounding safe access to medical cannabis. Canadian compassion club operators were invited to present before the Senate Special Committee on Illegal Drugs, which made the following recommendations in Chapter 9 of their final report:

• Measures should be taken to support and encourage the development of alternative practices, such as the establishment of compassion clubs;

• The practices of these organizations are in line with the therapeutic indications arising from clinical studies and meet the strict rules on quality and safety;

• The qualities of the marijuana used in those studies must meet the standards of current practice in compassion clubs, not NIDA standards;

• The studies should focus on applications and the specific doses for various medical conditions;

• Health Canada should, at the earliest possible opportunity, undertake a clinical study in cooperation with Canadian compassion clubs. [15]

Additionally, Hilary Black (Founder of the BCCCS) and I were invited to represent compassion clubs in a presentation before the OCMA Stakeholder Advisory Committee in the Spring of 2003, and made a number of recommendations to improve the federal program, including the decentralization of medical cannabis access in Canada, and the need to have the end-user costs of this medicine covered by provincial registries [16]. In a broader stakeholder consultation organized by the OCMA in 2004, representatives of the BCCCS and VICS produced and distributed a document titled "Roadmap to Compassion; The Implementation of a Working Medical Cannabis Program in Canada" [17], which examined many of the ongoing issues restricting medical cannabis access through Health Canada's program, and set out a 12-month timeline for the decentralization of this federal policy and practice. In a section titled "Potential Concerns with a Decentralized Program", the authors respond to one of the key objections to decentralization and community-based access to medical cannabis, Canada's oft-cited international treaty obligations:

In the past, Health Canada has implied that the decentralization of this program is restricted by our international treaty obligations, the most significant of which are the Single Convention on Narcotic Drugs [(1961)], the Convention on Psychotropic Substances [(1971)] and the relevant portions of the United Nations Convention against Illicit Traffic in Narcotic Drugs and Psychotropic Substances [(1988)]. According to section (c) of the original 1961 treaty, a signing country has the right to produce any drug or substance so long as its use and distribution is: "Subject to the provisions of this Convention, to limit exclusively to medical and scientific purposes the production, manufacture, export, import, distribution of, trade in, use and possession of drugs." In other words, there should be no doubt that the trade, use and possession of drugs for medical or scientific purposes is permitted by the terms of this Convention.

The report concludes that "the future of a successful medicinal cannabis program in this country should focus on the distribution model that has already proven itself to be safe and successful: not-for profit distribution by community-based compassion societies". Health Canada has yet to acknowledge the experience and expertise residing in the compassion clubs, and refuses to consider the incorporation of this successful model into its medical cannabis access program.

Canada's compassion clubs and societies provide over 11,000 critically and chronically ill Canadians access to a safe supply of cannabis within an environment conducive to healing. Since these organizations have developed policies reflective of their local socio-political environment, there can be some significant variation in the scope and quality of services offered by these dispensaries. The VICS and BCCCS have recently attempted to address these operational differences by introducing a set of regulations based on the best-practices of these organizations titled "Guidelines for the Community-Based Distribution of Medical Cannabis in Canada" [18]. This document, released at the 2006 International Harm Reduction Association Conference in Vancouver, addresses the rights of both dispensaries and their clienteles, is designed to inform both local communities and compassion clubs of the roles and responsibilities of such organizations, and aims to:

1. Provide a base-standard for self-regulation of dispensaries based on current best practices in Canadian compassion clubs;

2. Support medical cannabis dispensaries in providing a high standard of care that clients can and should expect;

3. Help both distributors and end-users achieve maximum safety and therapeutic potential within a setting that is conducive to healing;

4. Formalize the good reputation established by compassion clubs, thus ensuring those with medical need have continued access;

5. Promote an understanding of medical cannabis dispensary practices to all levels of government, the justice system, law enforcement, and community partners;

6. Allow for effective cooperation amongst dispensaries utilizing the same base-standards of operation.4

7. Organize participating dispensaries into a more cohesive voice for the legitimization and legal acceptance of community-based cannabis production, research and distribution. (Chapter 4)

These guidelines are an attempt to introduce more transparency and accountability into the compassion club model, and they have been endorsed by over 80% of Canadian compassion clubs. Additionally, they are being used in the development of similar regulations in Washington State, Rhode Island, and many California municipalities.

## 2. Health Canada's Marihuana Medical Access Division

The federal government's own polling and research suggests that there are currently over 290,000 medical users in the province of British Columbia alone [19], and yet between the introduction of the federal medical cannabis program in January 1999 and September 2004, Health Canada only received 2838 applications from across the nation. According to the MMAD there were 757 registered medical users in Canada in September 2004, suggesting a rejection or drop off rate of nearly 75% in the first year of the program [20].

Although the Ontario Court of Appeals' Hitzig decision was supposed to ease access to Health Canada's "compassionate" program by reducing the bureaucratic burdens of the MMAR, the number of applicants per month declined steadily between April of 2002 and July 2005, with the OCMA only approving 47 of the 299 applications received between January and September of 2004. In fact, participation in the program shrank by 34 people in the three months between July and October of 2004, dropping from 781 down to 747 authorized users [21].

The problem of access was well-noted by the Canadian Senate Special Committee on Illegal Drugs in their final report on cannabis from 2002, which found that:

while a process that authorizes the possession and production of marijuana has been established in Canada, this has not ensured that cannabis is suitably available to those in need...we have come to the conclusion that the MMAR have become a barrier to access. Rather than providing a compassionate framework, the regulations unduly restrict the availability of cannabis to those who may receive health benefits from its use. [22]

According to this report, one of the main reasons for the small number of applicants to the program is reluctance by physicians to act as gatekeepers to medicinal cannabis. Citing a perceived lack of information on dosage, side effects, and alternate routes of administration to smoking, a number of provincial medical colleges, the Canadian Medical Association (CMA), and the Canadian Medical Protection Agency (which insures nearly 95% of Canada's physicians) have warned against the therapeutic use of cannabis, and have recommended that doctors not participate in the federal program. For example, a CMA press release dated July 9^th^, 2003, declares:

The CMA has consistently raised concerns about the lack of evidence-based decisions to support the Medical Marijuana Access Regulations," said Dr. Dana Hanson, President of the CMA. "Our unease over use of medical marijuana has been ignored in this new policy. Physicians should not be the gatekeeper for a substance for which we do not have adequate scientific proof of safety or efficacy [23].

Although the CMA's position is now more supportive of the program, these initial warnings were a particular deterrent for Canada's medical specialists, whose support was initially necessary for all applicants to the program that were neither terminally ill nor likely to die in the next 12 months, such as those suffering from MS, HIV/AIDS and hepatitis C (terminal patients only required the support of a single physician). In addition, specialists were simply not available in many smaller rural communities. When compounded by the bureaucratic hurdle of filling out a 29-page application that sometimes took in excess of 12 months for Health Canada to process, the challenges to participation in this program ranged from onerous to impossible for many potential applicants.

Health Canada officially amended the MMAR application process in 2005 to remove the requirement of a supportive specialist for most medical cannabis users. However, this new "simplified" application form was now 33 pages long, and potential applicants continued to face resistance from the medical community. The burden of this difficult application process is apparent in comparing the MMAD with the state-run Oregon Medical Marijuana Program (OMMP). Although both programs originated in 1999 and have similar medical requirements for registration, Oregon's simple two page application process has led to the registration of nearly 15,000 participants as of October 1^st^, 2007 – despite having a population one-tenth that of Canada [24].

The Canadian Senate Special Committee addressed this problem by suggesting that the proper role of the physician in this program should be to make a diagnosis of the patient's medical conditions or symptoms, after which "the patient should be authorized to use...cannabis if the condition or symptom is one where cannabis has potential therapeutic applications" [25]. Health Canada has yet to heed this advice or change its policy accordingly, so as of June 2007 only 1816 Canadians were benefiting from this federal program [26].

Additionally, potential and actual participants in the program have made Health Canada well-aware of their legitimate concerns by filing official complaints with the MMAD. An "Access to Information" request for copies of all "paper letters of complaint received by Health Canada or the OCMA regarding the federal cannabis program" resulted in over 2000 pages of documented complaints over the first six years of the program, which including problems accessing the federal program, unexplained bureaucratic delays in processing applications and yearly renewals, and criticisms of quality of the federal cannabis supply [27].

In a federally-funded report titled "Our Rights, Our Choice" that examined the human rights, ethical and legal challenges faced by people living with HIV/AIDS who choose to use medical cannabis, the Canadian AIDS Society found that although between 14 to 37% of people living with HIV/AIDS used cannabis to address their condition, many had faced hurdles accessing the federal program [28]. The CAS report states that:

...access to the federal program remains hindered by barriers such as a lack of awareness of the program's existence, mistrust in the government, misinformation about the program and difficulty in finding a physician to support their application. Thousands of seriously ill Canadians must therefore choose between breaking the law to use the therapy of their choice, or going without, which in many cases compromises their well-being and quality of life. (p.2)

## 3. The Canadian Institute of Health Research and the Medical Marihuana Research Program

Since the court-ordered implementation of a federal medical cannabis policy in 1999, Health Canada has actively promoted its program to encourage and fund studies into the safety and effectiveness of medicinal cannabis. With the launch of the Canadian Institute Health Research's (CIHR's) Medical Marijuana Research Program (MMRP) and the establishment of a 5-year, $7.5 million clinical research grant in 2001, Canada had a unique opportunity to become a world leader in cannabis therapeutics; however, the government's research agenda has proven to be rather anemic. Since the introduction of the MMRP, only three clinical research proposals have been approved for CIHR funding: a smoked-cannabis and chronic pain study initiated by McGill's Pain Center, an HIV/AIDS and appetite study by the Community Resource Initiative of Toronto (CRIT) at St. Michael's Hospital, and the recently announced COMPASS (Cannabis for the Management of Pain: Assessment of Safety Study), which is the first project of the CIHR Marijuana Open Label Safety Initiative (MOLSI).

In March of 2003 the OCMA abruptly cancelled the funding for the Toronto-based CRIT research project, despite having already distributed over $800,000 of a $2 million research grant for the study. This led to the resignation of Dr. Gregory Robinson – a physician and patient-representative living with HIV/AIDS – from the OCMA's Stakeholder Advisory Committee. He argued that Health Canada had created a "Catch-22" by insisting on clinical evidence before approving cannabis as a medicine, but then thwarting the clinical trials needed to gather such evidence. As Robinson stated in his resignation letter to then Health Minister McLellan: "I no longer have faith in your ability to understand compassion for seriously and chronically ill patients," adding that "as an AIDS patient, each moment is valued so much at this time in my life. My continuing commitment to the advisory committee would only be a waste of my time and advice" [29].

Likewise, the $260,000 McGill chronic pain and smoked cannabis clinical study – which was approved in 2001 – has suffered delays largely due to bureaucratic problems in accessing a suitable supply of research cannabis from Health Canada. And although Health Canada announced in December 2004 that the large-scale, multi-center MOLSI study was finally underway and in its initial recruiting stage, very little information is available in regards to this research project, and no results have been made public to date.

In June 2004, the CIHR quietly posted a notice indicating that funding for the MMRP was "suspended until further notice" [30]. Louise Dery, a Senior Strategic Science Advisor with the Office of Controlled Drugs and Substances, indicated that with no guarantees of continuing funding past 2006, the OCMA would not accept any more requests for funding until at least early 2005.

However, in September 2006, the ruling Conservative party announced that it was cutting $4 million earmarked for the MMRP, effectively terminating this program and ending all federal financial support for medical cannabis research in Canada. As a result, Health Canada's initial commitment to a five year, $7.5 million dollar research plan has in fact been reduced to a three year, two-study initiative.

A few Canadian compassion clubs have attempted to remedy this paucity of research by designing and implementing their own studies. Since 2001, the Vancouver Island Compassion Society (VICS) and the British Columbia Compassion Club Society (BCCCS) have initiated peer-reviewed, university-associated research into the effects of cannabis on both hepatitis C (with the University of California, San Francisco), and nausea and pregnancy [31]. In addition, the VICS was awarded a $50,000 grant from the U.S.-based Marijuana Policy Project to undertake the first high potency, smoked cannabis and chronic pain double-blind clinical study in North America. This study has received Institutional Review Board approval, but Health Canada approval is still pending. The VICS has also officially participated in federally-funded research, including a CIHR-funded sociological examination of the patrons of compassion clubs, and the Canadian AIDS Society research project mentioned earlier in this paper. This CAS report states that:

...it is critical that clinical research be conducted, otherwise the federal medical cannabis program will remain a special access program rife with unnecessary regulatory and bureaucratic barriers...research can be greatly enhanced by involving community groups or organizations such as AIDS service organizations or compassion clubs, from the development of the research protocol to the dissemination of results from a clinical trial. [32].

## 4. Health Canada's Production and Supply Policy and Practice

In December 2000 Health Canada awarded a five-year, $5.7 million contract for the production of a domestic supply of research-grade cannabis to Prairie Plant Systems (PPS), a Winnipeg-based company that proposed to grow the plants at the bottom of a former zinc and copper mine in Flin Flon, Manitoba [33].

This single-source production plan has been a source of much controversy ever since Health Canada reluctantly began the distribution of its product, and as of June 2007 there were only 356 authorized users purchasing their cannabis from PPS, which is less than 20% of Canada's authorized medical cannabis users. By comparison, 1288 of the 1816 medical cannabis users authorized through the MMAD have chosen to produce their own supply of cannabis [34].

Initial concern over this production contract began with investigations into the physical location of the PPS facility. According to research conducted by independent monitoring groups, as well as Environment Canada and Natural Resources Canada, high levels of heavy metal contamination are detectable in air, water and soil samples for over 100 square kilometers around the Flin Flon mine, which is the result of over 80 years of extensive mining and smelting in the area [35]. When concerns over the potential for heavy metal contamination were raised by end-users and advocacy groups like Canadians for Safe Access, Health Canada spokesperson Jirina Vlk responded by suggesting that the levels of heavy metals in the federal cannabis supply were "similar to what one finds in Canadian tobacco and are well within allowable limits." However, when asked what the allowable limits for tobacco were, she conceded that there are currently no Canadian standards limiting heavy metal content in either tobacco or cannabis [36].

The potency of the government-contracted PPS cannabis has also been called into question. In June 2004, Canadians for Safe Access commissioned tetrahydrocannabinol (THC) testing of the PPS product through the Quebec Institute of Public Health Toxicology laboratory. The results showed the product to be under 6%THC, rather than the 10% claimed by Health Canada [37]. In fact, according to a series of 23 Gas Chromatography Mass Spectrometer (GCMS) tests commissioned by Health Canada and conducted by three different federally-licensed laboratories on the cannabis distributed to authorized medical users between August 2003 and May 2004, the THC content of this product never measured above 7.2%, averaging just over 6.2%THC, well below the 10% labeled on the product [38]. No contrary test results have ever been released by the federal government.

A recent study by Ware, Ducruet & Robinson suggests medical cannabis users can readily and reliably distinguish between cannabis products based on THC content, humidity, grind size and smoking characteristics [39]. Comparing four different products – including the PPS cannabis sent to authorized users until May 2004 – the study determined the government-approved cannabis was 6.6%THC rather than the "10% THC blend" suggested by Health Canada. The study found that "Product 3...which had been originally shipped by Health Canada to authorized patients was rated poorly by the [8] subjects in this study", with end-users finding it "worse than their usual cannabis". In May 2004 Health Canada began to distribute a more potent cannabis product to end users containing 12%THC, and additional improvements including increases in grind size and humidity have taken place in subsequent batches. However, the lack of strain selection is a concern that remains unacknowledged and unaddressed by Health Canada.

Additionally, results from biological testing obtained through Health Canada continue to indicate high levels of mold and biological impurities prior to gamma-irradiation. In six microbiological tests from 2004, the levels of aspergillus and penicillium mold averaged 536.66cfus (colony forming units) and 3872.5cfus respectively [40]. According to the U.S. Food and Drug Administration Center for Food Safety and Applied Nutrition, both Aspergillus and Penicillium mold produce dangerous mycotoxins like aflatoxin and ochratoxin that cannot be destroyed by gamma irradiation [41,42]. According to Dr. Dave Abramson, a mycotoxicologist with Agriculture and Agri-Food Canada, the only way to guarantee that a commodity is free from a specific mycotoxin:

...is to sample the crop in a representative manner, and then perform a quantitative assay following a published and validated procedure. Depending on the crop and place of origin, specific assays for several mycotoxins would be necessary to ensure product safety. [43]

When asked specifically about microbial contamination and inhalation as a route of ingestion, Dr. Abramson states that "environmental studies have shown that all mycotoxins pose a very significant inhalation hazard," adding "there is some evidence that certain mycotoxins would survive the high temperatures associated with smoking, and remain potent in the vapor phase [43]."

Although Health Canada states that "the dried marihuana product meets Canadian requirements applicable to Natural Health Products (NHP) [44], these regulations require that all manufacturers of herbal medicines test for the presence of mycotoxins. However, neither Health Canada nor PPS performed or commissioned any such testing on the federal cannabis supply until May 2005 [45], despite having distributed this product to hundreds of critically and chronically ill Canadians for over 20 months. Although the testing that eventually took place revealed that mycotoxins levels on all PPS crops were below detection, the MMAD's misleading or unsupported statements in regards to the potency, safety standards and actual testing of this cannabis supply has caused justifiable concern and mistrust amongst both authorized users and advocacy groups.

Additionally, the biological decontamination technique used on the federal cannabis crop may turn out to be a health concern in its own right. Gamma irradiation is a highly controversial method of decontamination, and this researcher has been unable to find any studies assessing its safety on smoked or inhaled materials anywhere in the world. Research shows that along with molds and bacteria, it destroys beneficial terpenoids like myrcene, caryophyllene and linalool [46,47] that have known therapeutic properties and which may improve the bioavailability of some cannabinoids [48,49].

Further anecdotal evidence of the inadequacy of the government cannabis supply came from the actual end-users of this product, including longtime authorized user Jim Wakeford who stated to the press that the first batch of PPS cannabis was "totally unsuitable for human consumption" [50]. Out of the 93 people who had ordered the initial PPS product as of March 2004, nearly 30% returned it to Health Canada [51]. Although Health Canada cites much lower return rates for subsequent batches of cannabis, this may be the result of changes to their refund policy. Initially, dissatisfied end-users could return what was left of their package to Health Canada/PPS for a partial re-imbursement. However, under the current policy refunds are only offered for unopened packages, therefore if end-users open the sealed foil pouch in order to sample the PPS cannabis, they cannot return the product for a refund.

In February 2005 Canadian Press reported that according to Health Canada, 127 of the 278 patients ordering PPS cannabis from the government at the time were in arrears, for a total of $168,879 in unpaid medical cannabis bills. Health Canada responded by sending collection agencies after those in arrears for more than 180 days, cutting off at least 19 authorized users from ordering medical cannabis from the nation's only legal supplier, and forcing these critically and chronically ill Canadians to resort to accessing their medicine from illicit sources [52]. In light of this, the Canadian AIDS Society has recommended that the federal government give immediate consideration to "mechanisms for reimbursement of the costs of medical cannabis for seriously ill Canadians" [53].

However, this significant impediment to medical cannabis access remains unaddressed, and an internal Health Canada report titled "Audit of the Management Processes for the Medical Marihuana Program" from March 13, 2007 shows that although the federal government is aware of the inability of many authorized users to pay for the cost of this medicine, the federal response has been to increase pressure by ceasing shipments after 30 days:

The Departmental Audit Committee Risk provided support in 2005 to clarify the supply policy for Marihuana for Medical Purposes and cease shipment to clients in arrears. Senior Management was aware that a client's file may eventually be sent to collections. The Programme Management Committee of DSCSP recently approved further refinement of the supply policy to cease shipment to clients in arrears more than 30 days [54].

Although the long-term impact of this policy change is unclear at this time, the level of debt by end-users of the federal cannabis supply has increased dramatically since 2005. As of April 30^th ^2007, 229 authorized users who had ordered this cannabis supply had received notices that their accounts were in arrears, representing $297,920 in unpaid debt [55]. Upon receipt of these notices authorized persons are only allowed to order one more shipment of cannabis before being cut-off from Canada's only legal supply for non-payment. Additionally, 29 accounts have already been sent to collection agencies, cutting off these critically and chronically ill Canadians from their supply, and adding unnecessary stress to their health and well-being.

This is particularly vexing in light of recent information revealing that Health Canada significantly increases the retail price of this product as compared to the actual wholesale cost. An examination of the production and supply contract extension between Health Canada and PPS covering the period from January 2006 to September 2007 titled "A Review of the Cannabis Cultivation Contract Between Health Canada and Prairie Plant Systems" suggests that the federal government pays PPS $328.75 per kilogram of cannabis (approximately $10 per ounce), but then charges patients $150 per ounce, constituting a 1500% markup on a product that has already been paid for by Canadian taxpayers through an ongoing six year and nine month contract agreement totaling $10,278,276 [56]. The report goes on to compare the approximate cost of producing and supplying medical cannabis user through PPS/Health Canada vs. the BCCCS for the fiscal year of November 2005-October 2006. According to Table 1, the British Columbia Compassion Club Society supplies a safe source of cannabis to over 3000 sick or suffering Canadians for approximately the same yearly costs as Health Canada currently spends on the PPS production contract to supply just over 700 end-users, meaning that the total operating cost per person supplied through Health Canada/PPS is $3889.49 per person vs. $739.25 through the BCCCS. The report came to the following conclusions:

**Table 1:** Cost Comparison of PPS Contract Extension for Oct 2006-Sept 2007 to BCCCS Costs for Fiscal year of November 2005-October 2006

*Program Variables*	**Health Canada**	**BCCCS**
Number of Persons Accessing Product	700(a)	3000
Cost of Program	$2,722,643	$2,217,772(b)
Total Cost/Person	$3,889.49	$739.25
Cost of Cannabis	$547,627(c)	$1,299,409(d)
Quantity of Cannabis	778 kg(e)	262 kg
Cost of Cannabis/kg	$328.75/$1144(f)	$4959.57
Cost of Cannabis/Person	$782.32(g)	$433.13
Usable Percent of Cannabis	63% (h)	97% (i)
Cost of Unusable Cannabis	$202,622(j)	$43,345
Price to Patients/kg	$5000	$8000
Mark-Up on Price	1500%	66%
Operating Costs(k)	$2,175,016	$718,948(l)
Operating Cost/Person	$3,107.17	$239.34
Operations as Percent of Total Cost	80%	32%
Ratio of Operating Cost to Cannabis	4:1	1:2

(a) 350 license holders and 350 COMPASS study participants. Compass study ending Dec 31, 2007.

(b) Includes all costs directly related to provision of cannabis as well other cannabis products (i.e. hashish, tinctures and baked goods), and smoking implements. Does not include costs directly related to provision of other natural health care services also provided by the BCCCS.

(c) $138,075 for 420 kg plus $409,552 for 358 kg.

(d) Does not include costs of hashish or other cannabis products.

(e) Bulk product.

(f) $328.75/kg for 420 kg and $1144/kg for amounts of 240–358 kg above 420 kg.

(g) $197.25 for the 420 kg, and $585.07 for the 358 kg.

(h) According to PPS, 45% of bulk product is usable (see footnote 20)

(i) Loss of usable product purchased by the BCCCS is due to moisture loss and stems. Product must meet our manicuring standards as a condition of purchase.

(j) Using the conservative number of 37% unusable cannabis. At 55%, this would total $310,195.

(k) For these purposes, defined as all costs above cost of cannabis, including packaging and processing orders.

(l) Does not include wages related to provision of other natural health care services, however does include rent and utilities related to those services.

The 1500% mark-up on the cannabis charged to patients highlights the risk of Health Canada creating a monopoly over supply. Health Canada is requiring taxpayers and medical cannabis patients to fund inefficient practices, capital upgrades, and equipment for a private contractor. Instead of providing affordable medicine to those in need, Health Canada has chosen a policy and program that seemingly creates a windfall for one monopoly supplier to the detriment of patients and taxpayers. While community-based medical cannabis dispensaries provide a cost-effective alternative to Health Canada's centralized monopoly for cultivation and distribution, the end-cost to patients still remains problematic. The cost of cannabis for those in medical need must be covered under Canada's universal health care system as it is for other medicine. Canada's critically and chronically ill deserve the most affordable and highest quality care.

Furthermore, although nearly 80% of authorized users choose to cultivate their own supply, Health Canada has stated intentions to remove the right of patients to cultivate their medicine or to nominate someone to do so for them [57]. The Canadian AIDS Society found that only 1.7% of the people living with HIV/AIDS whom they consulted obtained their cannabis from Health Canada, compared to 35.9% who obtained it through compassion clubs:

Considering the current public attitude towards the government's cannabis, the fact that the government only provides one strain of cannabis to authorized persons, and the government's expressed intention to eventually phase out licenses to produce, we are concerned that people living with HIV/AIDS will have to continue to break the law to supply themselves with cannabis for their medicinal purposes...offering only one legal source and only one strain of cannabis for distribution to authorized Canadians may not be a constitutionally adequate alternative to the diverse supply currently available to them through license to produce, unauthorized compassion clubs, or within the black market [58].

This is supported by preliminary results from a survey study titled "Quality of Service Assessment of Health Canada's Medical Cannabis Policy and Practice" showing that only 8% of the 90 respondents – a sample size that represents over 5% of legally authorized users in Canada – currently order cannabis from Health Canada, and on a numeric scale from 1 to 10 – with 10 being "Excellent", and 1 being "Very Poor" – 76% of the respondents who had tried the Health Canada cannabis ranked it as being either a 1 or 2 [59].

Additional preliminary data from this study show that over 92% of respondents find that not all strains are equally effective at relieving their symptoms, and 97% say that they would prefer to obtain cannabis from a source that has a "large selection of different strains" rather than a single product. Finally, over 90% state that they'd prefer to purchase cannabis from a source that offers many different forms of ingestion, and given the option, over 81% of respondents would chose organic methods of cultivation for their medical cannabis supply. Unfortunately, Health Canada's current supply policy and practice has been unable or unwilling to address many of these end-user issues, leaving medical users little choice but to obtain their medicine from the black-market or from Canada's network of community-based dispensaries.

## 5. Community-Based Alternatives to a Centralized Medical Cannabis Program

"As far as the distribution of marijuana to qualified users is concerned, the government might consider creating properly regulated distribution centres or licensing compassion clubs, as proposed in the recent *Report of the Senate Special Committee on Illegal Drugs: Cannabis*."

- Ontario Supreme Court Judge Lederman (Hitzig v. the Queen, January 2003)

The Canadian Senate Special Committee on Illegal Drugs and the Ontario Court of Appeals have both suggested that Health Canada should seek to work with the nation's compassion societies with the goal of improving access to a safe supply of cannabis for legitimate users. Despite these recommendations and court orders, the MMAD has resisted opportunities to decentralize this program, forcing compassionate distributors and their suppliers to continue risking arrest and prosecution in an unregulated market. This risk is hardly theoretical; of the seven major clubs in Canada, more than half have been subjected to raids and arrests by law enforcement.

Although police raids continue to significantly disrupt safe access to cannabis by medical users, the federal prosecution of compassion clubs in Canada has been largely unsuccessful. Following a raid on the VICS in 2000 and a lengthy legal battle, B.C. provincial judge Higinbotham granted this author an absolute discharge for trafficking in recognition that:

...while there is no doubt that Mr. Lucas offended against the law by providing marijuana to others, his actions were intended to ameliorate the suffering of others. His conduct did ameliorate the suffering of others. By this Court's analysis, Mr. Lucas enhanced other people's lives at minimal or no risk to society, although he did it outside any legal framework. He provided that which the Government was unable to provide – a safe and high quality supply of marijuana to those needing it for medicinal purposes. (R. v. Lucas, July 5^th^, 2002)

There are some clear philosophical differences between the federal program and the work of compassionate distributors (Table 1). Most of Canada's compassion clubs focus on holistic care and harm-reduction, and many have used their unique experience and expertise to enhance consumer options in cannabis-based therapies. So while Health Canada currently offers medical patients a single strain of pre-ground raw plant material, compassion clubs distribute numerous different symptom-specific strains and offer many alternative methods of ingestion to smoking, including edibles, oils, tinctures, vaporizers, and sublingual sprays.

After an intensive investigation of medical cannabis access in Canada conducted by the Canadian AIDS Society, their final report supported the licensing of compassion clubs, stating:

...we favour a not-for-profit, community-based model of distribution of medicinal cannabis and its related services...these organizations also offer a number of different strains and alternatives to smoking, and are currently serving more than 10,000 Canadians...we recommend that the government authorize compassion clubs that meet defined operational standards and recognize them as legal dispensaries for medicinal cannabis [60].

Despite the significant potential to decrease the overall operational costs of this federal program, and to increase efficiency and end-user satisfaction through community-based access, Health Canada's Marihuana Medical Access Division continues to resist regulating and licensing Canada's compassion clubs and societies.

## 6. Discussion and Conclusion

Since 1999 the Canadian government has spent over $30 million in funding for the research, production and distribution of medicinal cannabis [61], yet there is a growing body of evidence that Health Canada's program is not meeting the needs of Canada's medical cannabis patient community, and that it may actually be acting as an impediment to safe and timely access. As a result, the policies of the Marihuana Medical Access Division may be significantly limiting the potential individual and public health benefits achievable though the timely and effective therapeutic use of cannabis by sick and suffering Canadians.

Originally implemented to ensure legal access to medicinal cannabis for the critically and chronically ill who might benefit from its use, Health Canada's MMAD has instead limited participation in the federal program through obstructive and arguably unconstitutional regulations, and restricted options to a safe supply of raw cannabis and alternative methods of ingestions by entrenching a restrictive, non-beneficial monopoly on production and distribution. Despite Health Canada's insistence that more research needs to be conducted on the safety and effectiveness of cannabis, the government has only funded two clinical cannabis studies since 1999, and has recently terminated all other federal financing for this emerging area of health research. Disturbingly, over the last six years fewer than 1900 applicants have been able to negotiate the cumbersome bureaucratic obstacles allowing them to participate in this federal program, and preliminary results from a quality of service assessment of Health Canada's medical cannabis policy and practice show that over 72% of the study's respondents cited that they are "somewhat" or "totally unsatisfied" with Health Canada's program [62]. In response to these ongoing problems, Canadian Member of Parliament Libby Davies (Vancouver East), Senator Pierre-Claude Nolin, and the Canadian AIDS Society have all called for a performance audit of the MMAD by the Auditor General of Canada.

Meanwhile, Canada's compassion clubs and societies continue to supply cannabis to more medical users than the MMAD, to initiate and participate in more medical cannabis research than CIHR, and to produce a more varied cannabis supply than Health Canada; all at no cost to the nation's taxpayers. The following are five steps that could significantly improve medical cannabis access in Canada:

1. An immediate audit and review of entire federal medical cannabis policy and practice.

2. Cost coverage of medical cannabis by federal/provincial healthcare programs, and debt forgiveness for current authorized users.

3. The re-implementation of the Ontario Court of Appeals changes to the MMAR in order for it to meet its minimum constitutional obligations.

4. The decentralization of the program to allow for immediate access and legal protection with a health care practitioner's recommendation.

5. The development of a cooperative relationship between Health Canada and compassion clubs to improve access, increase supply/ingestions options, and jump-start research into the therapeutic potential of cannabis.

While the fate of the federal program remains unclear, evidence suggests that any future success will likely depend on the government's ability to better assess the concerns and needs of the nation's critically and chronically ill, to promote and fund an expanded clinical research agenda, and to work in cooperation with Canada's established network of community-based medical cannabis compassion clubs in order to address and remedy the ongoing issue of safe and timely access to this herbal medicine.

## Competing interests

The author is the founder and director of the Vancouver Island Compassion Society, and receives a salary from this organization for research, communications and administrative work. The author is also the founder of Canadians for Safe Access and continues to do work on behalf of this non-profit organization, but as an unpaid volunteer.
